# Robust Optimal Design of Experiments for Model Discrimination Using an Interactive Software Tool

**DOI:** 10.1371/journal.pone.0055723

**Published:** 2013-02-04

**Authors:** Johannes Stegmaier, Dominik Skanda, Dirk Lebiedz

**Affiliations:** 1 Center for Analysis of Biological Systems (ZBSA), University of Freiburg, Freiburg, Germany; 2 Institute for Applied Computer Science (IAI), Karlsruhe Institute of Technology, Karlsruhe, Germany; 3 Institute for Numerical Mathematics and Ulm Center for Scientific Computing (UZWR), University of Ulm, Ulm, Germany; University of Catania, Italy

## Abstract

Mathematical modeling of biochemical processes significantly contributes to a better understanding of biological functionality and underlying dynamic mechanisms. To support time consuming and costly lab experiments, kinetic reaction equations can be formulated as a set of ordinary differential equations, which in turn allows to simulate and compare hypothetical models *in silico*. To identify new experimental designs that are able to discriminate between investigated models, the approach used in this work solves a semi-infinite constrained nonlinear optimization problem using derivative based numerical algorithms. The method takes into account parameter variabilities such that new experimental designs are robust against parameter changes while maintaining the optimal potential to discriminate between hypothetical models. In this contribution we present a newly developed software tool that offers a convenient graphical user interface for model discrimination. We demonstrate the beneficial operation of the discrimination approach and the usefulness of the software tool by analyzing a realistic benchmark experiment from literature. New robust optimal designs that allow to discriminate between the investigated model hypotheses of the benchmark experiment are successfully calculated and yield promising results. The involved robustification approach provides maximally discriminating experiments for the worst parameter configurations, which can be used to estimate the meaningfulness of upcoming experiments. A major benefit of the graphical user interface is the ability to interactively investigate the model behavior and the clear arrangement of numerous variables. In addition to a brief theoretical overview of the discrimination method and the functionality of the software tool, the importance of robustness of experimental designs against parameter variability is demonstrated on a biochemical benchmark problem. The software is licensed under the GNU General Public License and freely available at http://sourceforge.net/projects/mdtgui/.

## Introduction

The investigation of biochemical processes inherent to any biological systems such as bacteria, plants, animals or even humans is an important component of current systems biology approaches. To simulate such dynamic processes *in silico*, mathematical modelling based on ordinary differential equations (ODEs) represents a powerful and broadly used tool [1]. Although these models mostly do not represent the exact biochemical mechanisms of the investigated phenomena, they are useful to get deeper insights into the underlying processes or allow to make proposals for future experiments [2], [3]. The identification of suitable hypothetical models that simultaneously perform well on acquired measurements of different experiments is a challenging task. Of course, if a model is over-parametrized, it is possible to perfectly reproduce observed measurement data while concurrently loosing the generalization capabilities of the model [4]. On the other hand, if the number of model parameters is not sufficient, the model might be unable to fit the data at all. This means that a good model should be a trade-off between being as simple as possible while maintaining a good approximation of observed measurement data [5].

Frequently, several models perform equally well on an initial measurement data set. Instead of performing a huge number of different experiments to determine the appropriate hypothesis, simulating and re-designing experiments *in silico* before moving back to real experiments is a reasonable approach. It is therefore desirable that newly designed experiments allow to evaluate the appropriateness of the investigated models and possibly discard *incorrect* models. This process is also referred to as optimal experimental design for model discrimination or model selection.

Current methods for model discrimination range from exhaustive search methods [6] over probabilistic approaches [7] to optimization based approaches [6], [8], [9]. The approach considered in this work is based on reformulating the calculation of new experimental designs as a constrained nonlinear optimization problem. Therefore, an optimality criterion is maximized with respect to the involved experimental design variables such as the arrangement of measurement time points, the initial species concentrations, the experimental duration and external perturbations to the system. The concentration time courses of the participating species are attained by efficiently solving an initial value problem for the ODE based formulation of the biochemical reaction equations using numerical integration. Derivatives are generated using algorithmic differentiation which in turn allows to make use of efficient derivative based optimization algorithms [10]. The interior point optimization algorithm Ipopt is used to solve the constrained nonlinear optimization problem [11], [12].

Another aspect that arises upon incomplete *a priori* knowledge of certain involved parameters is the calculation of robust optimal designs. The intention of robust optimal designs for model discrimination is to maintain the power to select between several hypothetical models, even for the most inappropriate parameters that can be observed in a given setup. This is achieved by re-estimating parameter values in addition to calculating the discriminating designs.

In this article we present a comprehensive extension to the previously presented command line tool ModelDiscriminationToolkit by Skanda and Lebiedz [13]. The extension comprises a convenient graphical user interface for the interactive analysis of biochemical models as well as an implementation of the robustification approach described in [10]. Initially, we provide the reader with a brief introduction to the discrimination approach presented in [10], [13], introduce the basic concepts of the robustification method and discuss its realization in the ModelDiscriminationToolkitGUI. The results section emphasizes the importance of robust experimental design and demonstrates the capabilities of the ModelDiscriminationToolkitGUI on two examples, including a realistic benchmark problem on a biochemical network in an artificial organism [6]. Finally, the work is summarized and an outlook on further work is given in the last section.

## Methods

### Model representation

A widely used approach to model biochemical reaction networks is their formulation as a set of coupled ordinary differential equations (ODE). The temporal concentration change 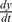 of a certain species 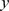 is defined as a function of the concentrations of the involved species with respect to the corresponding rate constants according to the law of mass action [14]. A comprehensive treatment of the modeling of biochemical processes using ordinary differential equations can e.g. be found in [14], [15].

As the models specified this way usually do not have an analytical solution, the equation system has to be solved numerically [16]. If the kinetic parameters of the reactions are not known in advance, they have to be estimated on given measurement data. By specifying the initial concentrations 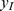 for all participating species, the ODEs can be solved using numerical procedures for solving initial value problems as described later. All models considered in this work are formulated as such a set of ordinary differential equations. The next sections show how to calculate new experimental designs for the discrimination of several plausible hypotheses formulated as ODE models.

### Model discrimination

The central goal of model discrimination is to calculate new experimental designs in such a way that the time courses of the rival model responses are maximally separated. Hence, an objective function is needed that evaluates the distance between the trajectories of the model hypotheses in a suitable way. In our case the objective function is derived by the Kullback-Leibler (KL) divergence, which is a non-symmetric measure for the distance of two probability density functions, as described in [17]. A detailed derivation of the adjusted optimization criterion as well as the statistical background can be found in [10], [13]. The general objective function for a discrete set of measurement points and possibly unequal variance functions 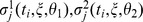 of the two models reads as follows:
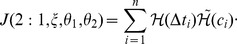


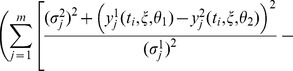
(1)

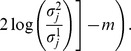



In Eq. (1), the sum of squared differences is scaled by the corresponding variance functions of the models. For the homoscedastic case, Eq. (1) reduces to the sum of squared differences between the responses of the two models. In Eq. (1), 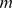 is the number of species, 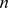 is the number of measurement time points and 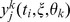 with 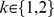 is the value of the time course trajectory of species 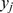 with 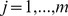 of the model 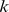. The variables 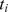 with 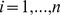 represent the measurement time points, 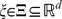 is a d-dimensional experimental design vector from the design space 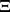 and 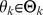 are the model parameters contained in the respective parameter spaces 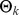. Note, the objective function in Eq. (1) is non-symmetric and therefore depends on the ordering of the models. In the following the ordering of the models in the objective function is not regarded for convenience.

An optimal experimental design 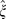 maximizes the objective function 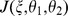 with respect to constant parameter vectors 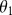 and 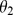. On the other hand, the objective function 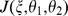 can also be used to determine *worst-case* parameters for a given design 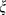 corresponding to most similar model responses. In this case, the experimental design is considered constant and the desired parameter vectors are subject to optimization. Of course, the criterion has to be minimized if worst-case parameters, which produce the most similar model responses, should be identified. The functions 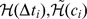 are weighting the model differences at a single measurement point depending on the time interval 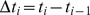 and the current species quantity perturbations 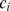 to the system. 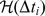 is used to avoid that time intervals fall below a predefined minimum measurement interval 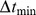, which models restrictions of the measurement method. In turn, the function 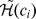 prevents simultaneously performed measurements and perturbations, which is demanded in some of the considered experiments. Mathematically this can be modeled by so called Heaviside functions that map from the real numbers to the set 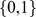.
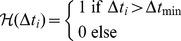
(2)

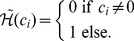
(3)The definition of the Heaviside functions can be found in Eq. (2) and Eq. (3). These Heaviside functions are not differentiable and therefore continuous differentiable approximations of these functions are needed in order to apply derivative based optimization algorithms. An approximation of these Heaviside functions based on parametrized hyperbolic tangent functions is given in [10], [13].

### Robust optimal designs

The parameters of the investigated models are usually estimated on available measurement data. Due to measurement inaccuracies, there is uncertainty about those parameters up to a confidence region representing a level of significance. Additionally, biological systems might involve intrinsically distributed parameters leading to the effect of distributed determinism as pointed out in [18], [19]. Hence, it is desirable to have new experimental designs with both great discrimination power as well as keeping the ability to distinguish between the models even for inappropriate parameter settings within some confidence region. These demands naturally lead to the problem of finding a worst-case optimal experimental design 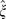 with respect to appropriate parameter spaces 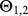 and thus 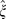 is referred to as robust optimal design. Mathematically, the problem of finding a robust optimal design 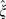 can be formulated as a max-min optimization problem in the following way:
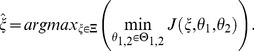
(4)Here, the experimental design vector 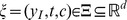 consists of the initial species concentrations 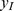, perturbations 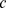 and an optimal arrangement of measurement time points 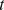. The set 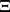 represents the space of feasible experimental designs and 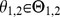 are the feasible model parameters for the current design 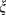.

We solve the problem stated in Eq. (4) iteratively by an outer approximation algorithm, see e.g. [20]. Each iteration 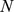 of the outer approximation algorithm consists of two phases. In the first phase worst-case parameters 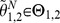 for the current optimal experimental design 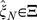 are re-estimated. (For the first iteration with 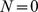, 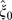 corresponds to the user supplied initial experimental design.) These worst-case parameters 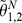 are used to augment a list of parameters 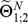 by 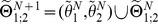. (For the first iteration with 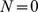, we set 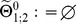.) In the second phase a new optimal experimental design 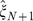 is calculated with the constraint that the design has to be optimal with respect to all parameter pairs in 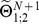 simultaneously, i.e.
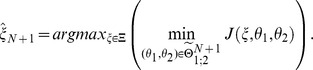
It can be shown [20], [21] under reasonable assumptions that 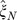 converges to a critical point 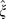 of the problem stated in Eq. (4), i.e. 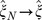 as 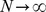. Thus, the values obtained for the objective functions 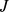 of the discrimination run in phase two and the parameter re-estimation in phase one of the outer approximation algorithm are converging to the same value. The absolute value of the difference of the objective functions, which we call the robustification gap 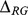, can be used as a stopping criterion of the max-min optimization problem:
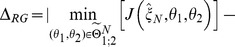


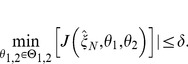
(5)
Eq. (5) shows the definition of the robustification gap as well as the stopping condition. The value for 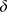 is usually chosen to be a small positive number, e.g. 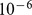, depending on the demanded accuracy and the problem under consideration. As soon as the robustification gap falls below a desired value, the discrimination algorithm can be stopped. To lower the chance of receiving only local worst-case parameters, several random initializations of the parameter estimation within a feasible range can be performed.

### The optimization problem

The full robust optimal design problem which is considered in this paper can be formulated in the following way:
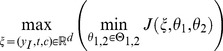
(6)subject to
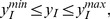
(7)

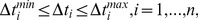
(8)

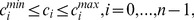
(9)

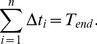
(10)The constraints for the initial species vector 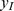 are necessary to avoid infeasible species concentrations, e.g. preventing negative concentration values. Since we demand that an experiment has to be performed in a fixed time span, the time intervals 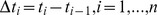, have to sum up to the fixed experimental duration 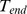 and have to lie in the range 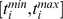. Finally, the constraints for the perturbations 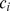 ensure that these values also lie in a reasonable range. The numerical realization and a more thorough mathematical formulation of this semi-infinite inequality and equality constrained optimization problem (SIECP, [20]) for the purpose of robust optimal design of experiments is given in [10], [21].

### Numerical tools

To solve the initial value problems for the ODE models, a backward differentiation formula (BDF) integrator is used and implemented in a multiple shooting setup [21], [22]. Derivatives needed by the optimization algorithm are calculated using the automatic differentiation software CppAD and the optimization problem is solved using an interior point approach implemented in the Ipopt package [11], [23]. Additionally, a so called homotopy method, which introduces new constraints on the parameter values slowly to subsequent optimization steps, is used to improve the convergence of the outer approximation algorithm [10], [21].

### The graphical user interface

**Figure 1:**
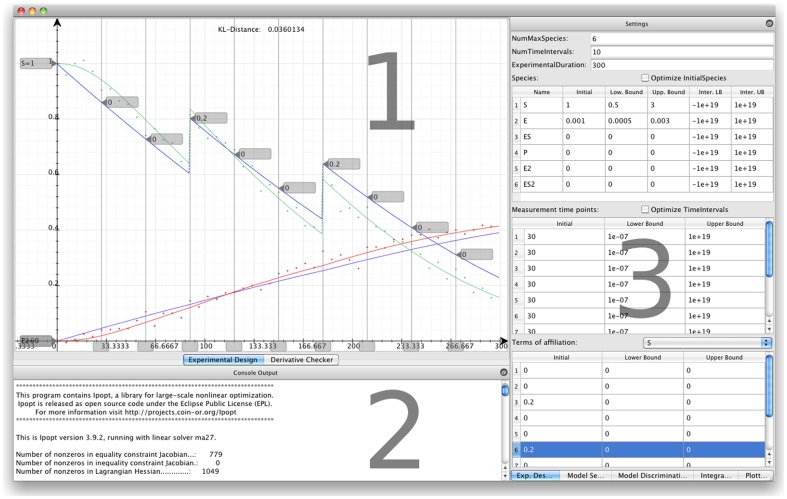
Screenshot of the graphical user interface. The main working areas of the GUI are labeled. The application comprises a plot window (1), a console output window (2) and an extensive settings dialog (3).

To make the functionality of the ModelDiscriminationToolkit available to non-programmers, a convenient graphical user interface, namely the ModelDiscriminationToolkitGUI, has been developed, delivering easy and interactive access to the abovementioned model discrimination techniques. An exemplary screenshot of the ModelDiscriminationToolkitGUI is given in Fig. 1. The application is separated into the three labeled areas. The plot window (1) displays all time courses and derivatives of the currently enabled species. It provides direct feedback of the optimization steps that are performed during the discrimination and can also be used to get a better understanding of the model behavior. Experimental conditions like initial species, measurement intervals and perturbations can directly be altered within the plotting window. The models are immediately simulated again and therefore different possible discrimination scenarios can be tested before starting the actual optimization routine. The console window (2) displays the output generated by the ModelDiscriminationToolkit and the optimization package Ipopt. Additionally, the intermediate experimental designs, worst-case parameters as well as the final results are printed there. The output format of the experimental design components such as time intervals and perturbations are given in MATLAB matrix notation and stored on disk in order to facilitate further processing of data. The settings window (3) can be used to adapt e.g. the experimental conditions, robustification properties, Ipopt parameters and integrator settings. A threaded implementation ensures that the GUI remains responsive to the user, e.g. allowing live update of the new experiment during optimization and a pause functionality for further investigations.

### Implementation details

The ModelDiscriminationToolkitGUI is developed in the C++ programming language using the Xcode IDE on Mac OS X for development. For the creation of the graphical user interface, the Qt API (4.7) by Nokia, which is freely available for non-commercial projects, is used [24]. The ModelDiscriminationToolkit requires the interior point optimization package Ipopt [11], [12] with the linear solver MA27 [25]. Throughout the presented implementation, the Ipopt version 3.9.2 is used. To fully exploit the parallel processing capabilities of modern hardware, the numerical integration is parallelized using POSIX threads. In addition to detailed installation instructions, getting started tutorials, Xcode project files and a Unix makefile, a template model file is provided to built and test the ModelDiscriminationToolkitGUI on different Unix based platforms. The application has successfully been compiled and executed on both Mac OS X 10.8 and Ubuntu 12.04.

## Results and Discussion

In this section we present the results of two exemplary discrimination problems that demonstrate the importance of the robustification approach as well as the capabilities of the ModelDiscriminationToolkitGUI. The first example deals with two models for enzyme catalyzed reactions. In the second example, the tool is applied to a realistic benchmark problem for model discrimination published earlier by Kremling et al. [6]. All calculations are performed using the current version of our software tool. The complete software package, all discussed models as well as the project files and result logs are offered for download on the project website http://sourceforge.net/projects/mdtgui.

### Hysteretic enzyme vs. simple enzyme

The enzyme catalyzed product molecule formation from certain substrate molecules is an ubiquitous process in living organisms. In this discrimination example we identify the appropriate enzyme model for a given set of measurement data. The most simple enzymatic reaction where a steady-state is reached directly after a short initial phase according to the reaction equations
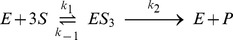
can be modeled by the following set of ODEs:
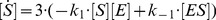
(11)

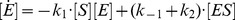
(12)

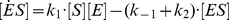
(13)

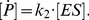
(14)


This model considers four species, namely substrate 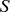, enzyme 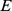, enzyme-substrate-complex 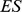 and the product 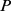. The substrate binds to the enzyme with a certain affinity, forming an enzyme-substrate-complex denoted by 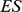 with a rate constant 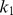. The rate constant 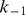 describes the dissociation of the enzyme-substrate-complex back into the single components and the rate constant 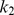 describes the decay of the enzyme-substrate-complex into free enzyme and the product. After a short phase of building the enzyme-substrate-complex, the concentration of the free enzyme approaches zero and the reaction enters a steady-state with constant turnover rate. After the substrate is depleted, the turnover rate reduces and the free enzyme concentration increases again [26].

Contrary to this, reactions catalyzed by so called hysteretic enzymes are characterized by a distinct lag phase before a steady-state is entered [27]. The RNAse and wheat germ hexokinase, for instance, are monomeric enzymes showing hysteretic behavior [26], [28]. This phenomenon, also known as kinetic cooperativity, can be explained by the slow transition model [26]. In contrast to cooperativity that can be observed when several subunits of a multimeric enzyme are interacting upon substrate binding, this model gives a plausible explanation for sigmoidal behavior of monomeric enzymes due to slow conformational changes. The enzyme exists in two conformational states 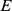 and 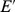, termed inactive and active, respectively, as they heavily differ in their catalytic activity. In absence of substrate the enzyme primarily exists in the inactive conformation. Binding of substrate molecules to the inactive enzyme initiates the conformational change and the enzyme is then able to work at a much higher efficiency until the substrate is depleted. The slow transition into the active conformation and a higher turnover rate of the active conformation, are the reasons for the sigmoidal shape of the substrate and product concentration curves plotted against the time. According to [27], these two enzymatic states can be modelled by the following reaction equations:
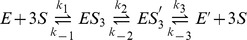


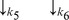


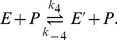
Again, these equations have to be reformulated as a set of ODEs for further numerical analyses:
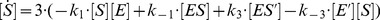
(15)

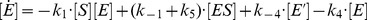
(16)

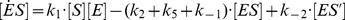
(17)

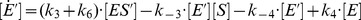
(18)

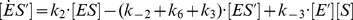
(19)

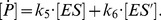
(20)


As soon as the rate constant 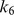 is significantly larger than 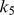 and the transition rate between 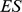 and 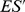 is sufficiently small, this model shows a clear sigmoidal shape for both the substrate and the product concentration over time. In this example three substrate molecules are used to form one product molecule, which is the reason for the factor 3 present in Eq. (11) and Eq. (15).

#### Data generation

**Figure 2:**
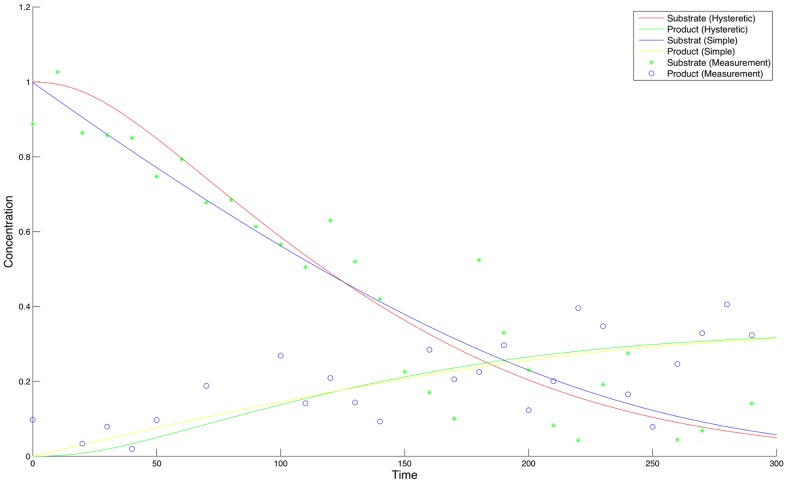
Substrate and product time courses of the simple model and the hysteretic model with estimated parameters. The values of one out of five measurements are shown as dots. A lack-of-fit test, which is performed on both models with the generated experimental data, ensures that none of these models can be discarded in advance. The depicted experiment is used as initial design of the discrimination approach.

**Figure 3:**
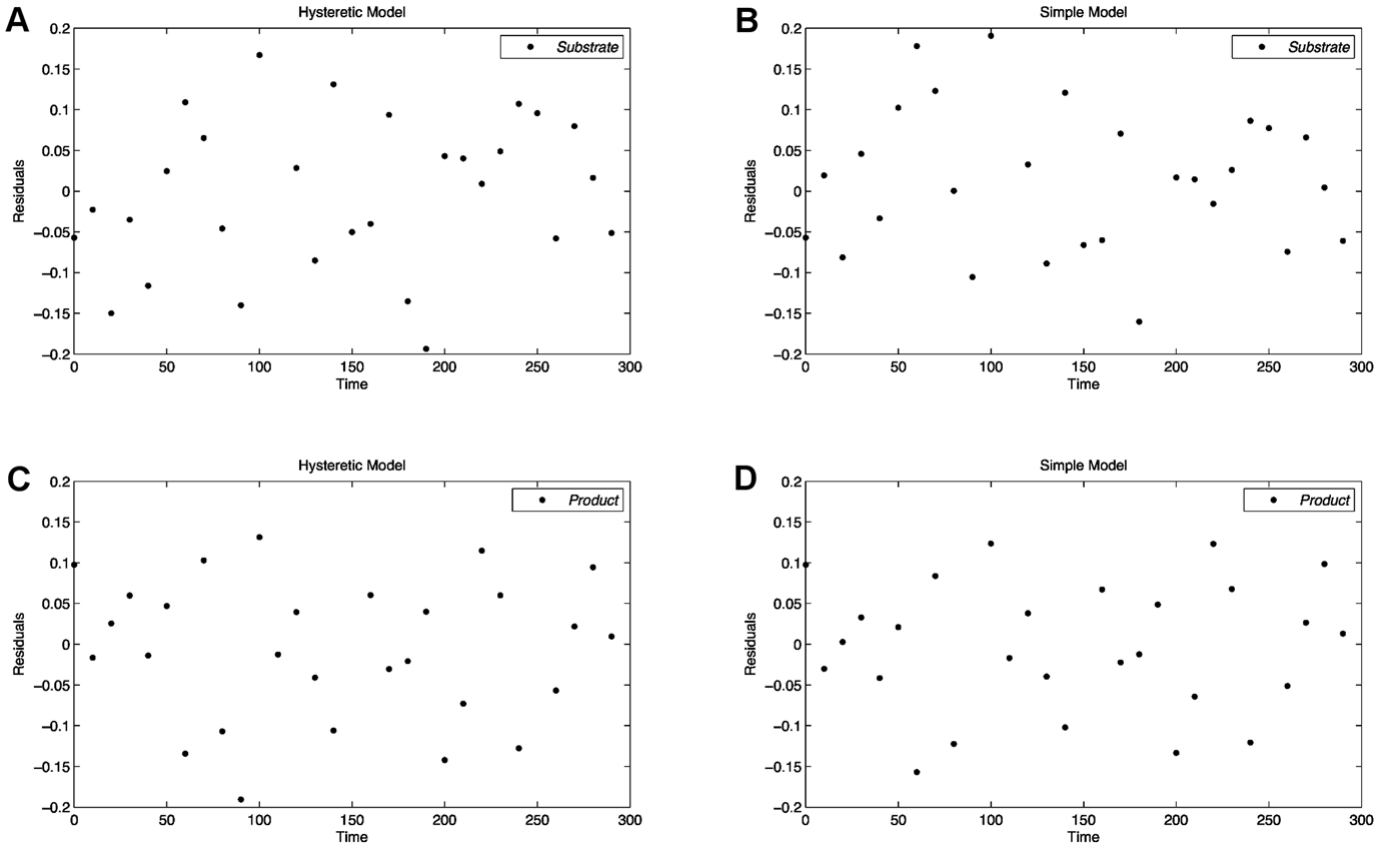
The residual vs. run time plots of the simple and the hysteretic model for the initial design. The images in the upper and lower part show the residuals of the substrate and the product curves, respectively. None of the plots shows a noticeable trend, which indicates a good fit of both considered models.

Due to the unavailability of real experimental data, artificial measurements are generated using the hysteretic model. The correct parameters listed in Tab. 1 are chosen such that a nice sigmoidal shape for both the substrate and the product concentrations over time is received. The hysteretic model is simulated over 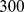 time units and measurements are performed every 10 time units. Initial concentrations for the substrate and the enzyme are set to 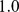 and 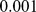, respectively, and the remaining species values are set to zero. No perturbations are performed during the initial experiment. Five independent random data sets are simulated using the hysteretic model with an additive normally distributed error term 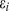 at measurement time point 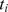 with standard deviation 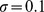 and mean 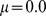. Each model-generated value 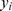 is altered to 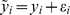 for all 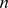 time steps in order to simulate measurement inaccuracies. As the standard deviation is equal for all measurements, the homoscedastic version of the objective function shown in Eq. (1) is used. For the further discrimination process the parameters of both models are assumed to be unknown and are simultaneously estimated on the generated measurement data. All parameters are initialized to one and subsequently fitted to the observed data in a least squares sense. Fig. 2 shows the substrate and product time courses of both models with estimated parameters, which serves as initialization of the discrimination method. To verify that none of the models can be discarded on the initial experimental data, a lack-of-fit test based on the decomposition of the regression curve residual variance is performed [29]–[31]. The test fails to reject the null hypothesis of an appropriate fit for both models (data not shown), i.e. it is not clear which hypothesis underlies the given set of measurements. Due to the absence of a noticeable trend of the residuals, a similar conclusion can be drawn based on the residual vs. run time plot shown in Fig. 3. Estimated parameters for the simple model and the hysteretic model are listed in Tab. 2 and Tab. 3, respectively.

**Table 1:** The correct parameters of the hysteretic enzyme model used for data generation.

Parameter:	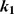	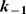	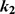	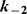	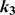	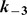	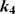	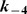	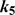	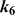
Value:	0.05	0.001	0.06	0.001	0.001	6.0	0.001	0.001	0.01	4.0

The correct parameters of the hysteretic model used for data generation. Parameters are manually selected using the GUI, such that a hysteretic shape is observable. Measurement data is produced by simulating the hysteretic model using the correct parameters at 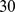 equally spaced sampling points. Sampled points are individually disturbed by use of additive Gaussian noise with standard deviation 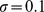 and mean 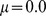.

**Table 2:** The estimated parameters for the simple enzyme reaction model.

Parameter:	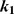	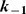	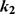
Value:	6.4086	0.0	2.0749

The estimated parameters for the simple enzyme reaction model. Based on measurements generated by a hidden hysteretic model with known parameters, the unknown parameters of the simple candidate model were fitted to five independently generated data sets in a least squares sense.

**Table 3:** The estimated parameters for the hysteretic enzyme reaction model.

Parameter:	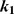	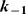	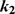	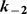	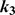	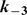	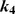	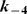	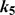	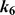
Value:	0.0708	0.0	0.0548	0.0	0.0	5.5020	0.0076	0.0	0.0	3.5715

The estimated parameters for the hysteretic enzyme reaction model. Based on measurements generated by a hidden hysteretic model with known parameters, the unknown parameters of the hysteretic candidate model were fitted to five independently generated data sets in a least squares sense.

The goal for the subsequent analysis is to determine an optimal experimental design that allows to reliably identify the correct hypothesis out of the two candidates. Additionally, it is demanded that the optimal design is robust to parameter variations of the simple model, i.e. even if the parameters of the simple model are re-estimated, a clear distinction of the models should be guaranteed.

#### Results and discussion

For this discrimination example the parameters of the simple model 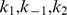 are subject to robustification. The box constraints for the parameters listed in Tab. 4 are estimated using the graphical user interface with random measurements generated by the correct model. Parameter values, where one of the curves of the simple model clearly exceeds the generated random measurement data are used as boundaries for the robustification. The parameters for the hysteretic model are fixed for the discrimination and are listed in Tab. 3. The initial substrate concentration is set to 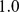 and constrained to the range 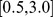. The initial enzyme concentration is set to 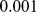 and restricted to the interval 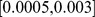. All other participating species are initialized to 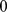 and the initial concentrations of these variables are not subject to optimization. The multiple shooting bounds are not restricted, i.e. the bounds are set to 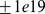. The experimental duration is set to 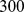 time units and divided into 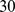 equally spaced time intervals. At ten equally spaced measurement points, perturbations of either substrate within a range of 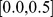 and/or enzyme within a range of 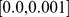 are allowed and subject to design. For a minimum time span between two measurement points the corresponding step function 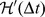 is set to a width 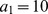 with the centre located at 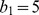. These step function settings ensure that measurement points below 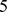 time units are disabled. In order to avoid simultaneous measurements and perturbations, the step functions 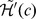 are set to the width 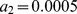 with centre at 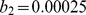. These parameters values ensure that the step function switches its value to zero as soon as a perturbation to the system occurs. The number of homotopy steps is set to 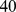 and the homotopy is started as soon as the robustification gap 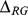 drops below 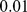.

**Table 4:** Initial parameter values and ranges for the simple model.

Parameter	Initial Value	Range
*k*_1_	6.40861	[0.5,100.0]
*k*_−1_	0.0	[0.0,100.0]
*k*_2_	2.07486	[0.5,30.0]

Initial parameter values and robustification ranges for the simple model. The box constraints are manually determined using the graphical user interface by comparing the model trajectories to measurement data generated with the hidden correct model.

**Figure 4:**
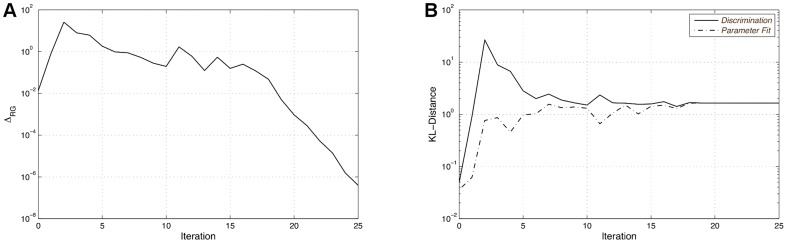
Robustification gaps and objective values. The robustification gap 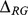 is displayed in the left part of the figure. The right part shows the corresponding objective values of the respective new designs (solid line) and the objective values of this design with re-estimated worst case parameters (dashed line). All values are plotted versus the corresponding iteration number.

For this setup the calculation requires approximately 24 hours on one core of an Intel Xeon X5460 CPU with 3.16 GHz. The robust optimal design is found after 25 iterations of the relaxation algorithm with a robustification gap 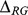 below the stopping criterion of 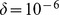. The robustification gaps versus the iterations are shown in part A of Fig. 4. In the beginning of the optimization, several jumps of the robustification gap value can be observed. These jumps clearly vanish the closer the algorithm converges to an optimum. Once the homotopy is started, here at iteration 20, no further jumps occur. This demonstrates the usefulness of the homotopy strategy, which stabilizes the convergence of the algorithm. Forcing the algorithm to reach such a small robustification gap is mostly not needed for practical applications, though. Since the accuracy of the used measurement method is the limiting factor, 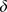 can be chosen relative to the measurement variance. Part B of Fig. 4 shows the values of the objective functions. The solid line represents the objective value of the new optimal design with respect to the parameter sets of the simple model found so far. The dashed line in turn represents the KL-divergence of the new design with re-estimated worst case parameters. It can be observed that even for designs that lead to huge KL-divergence values, e.g. after the second iteration, new parameters can be found that significantly decrease the model distances. After multiple iterations the objective values are approaching a constant value and the robustification gap finally drops below the stopping criterion. The optimum design proposed after the first few iterations of the algorithm uses the lowest possible initial species concentrations and nearly all perturbations are set to the maximum value. This clearly differs from the final result and causes the large jump of the objective values that can be observed after the second iteration.

**Figure 5:**
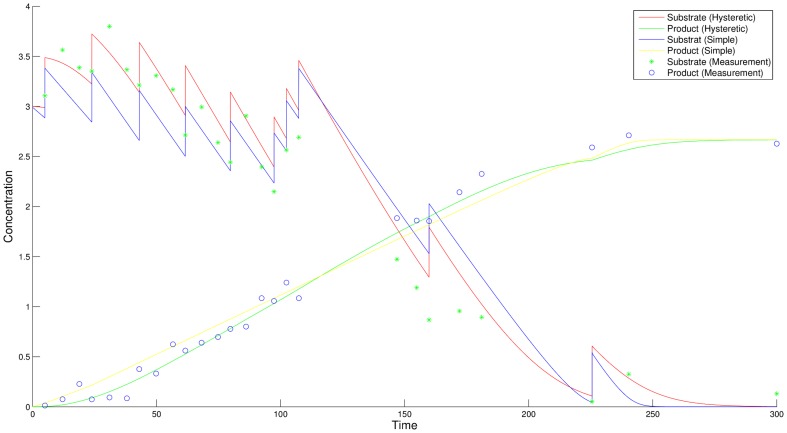
Time courses after discrimination. The time courses for substrate and product of the two models after discrimination compared to the experimental data. The worst case discrimination is shown. Other parameters in the feasible range lead to larger model distances.

In Fig. 5 the resulting robust optimal design is shown. The optimal measurement points of the new design are mainly moved to the beginning of the time horizon where the largest difference in the time courses of the species is observed. Four of the measurement points are omitted. Both free initial values are set to the maximum values of 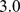 for the substrate and 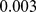 for the enzyme concentration. The substrate perturbations are set to 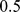 at each feasible point and the enzyme perturbations are only applied on the first two and the last possible perturbation point. Additional experiments (data not shown) yielded similar results for the discrimination with varied boundary conditions for the initial species concentration. The initial concentrations are raised to the upper bound and perturbations are applied in the beginning of the experiment.

**Figure 6:**
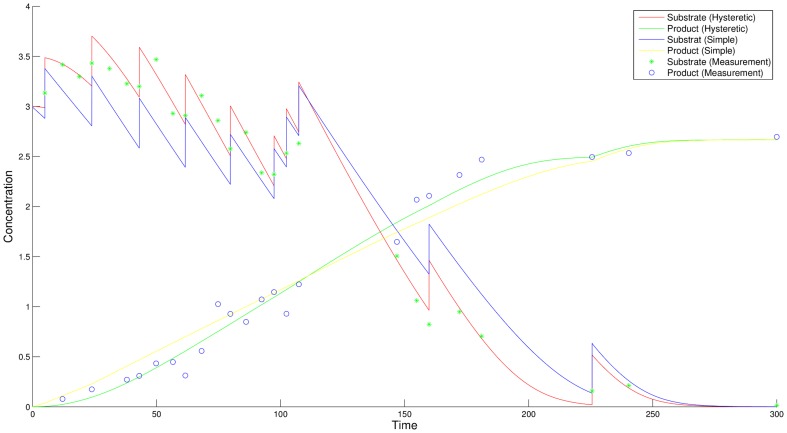
Time courses after discrimination with re-estimated parameters. The time courses for substrate and product of the two models after discrimination compared to the simulated experimental data. The parameters have been re-estimated using measurement data of the new design and the initial design. For a correct model it should be possible to determine parameter values that sufficiently fit all available measurements.

**Figure 7:**
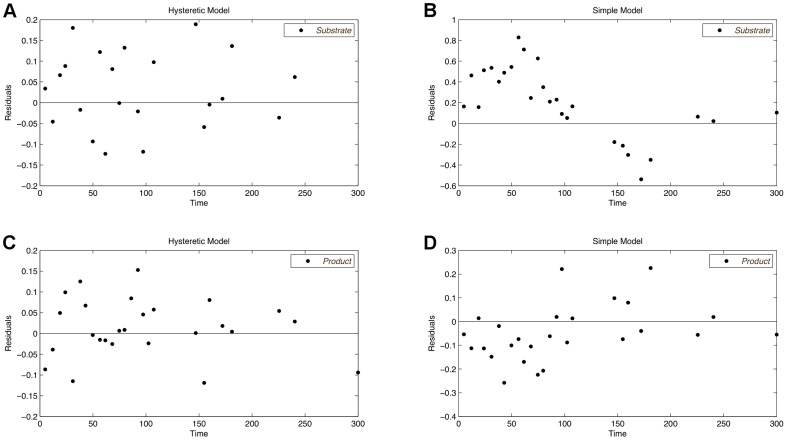
Residual plots after discrimination with re-estimated parameters for both models. The residuals of the simple model show a clear trend, which indicates the bad fit of the model even with re-estimated parameters (B, D). Contrary, the good fit of the hysteretic model is proofed by residuals being normally distributed around zero (A, C).

After the identification of the optimally discriminating design, none of the models is able to perfectly describe the data using the initially identified parameters. The parameters are re-estimated on measurements of both, the initial design and the new design. The re-estimated parameters for the two models are listed in Tab. 5 and Tab. 6, respectively. Simulating the models again with the re-estimated parameters, as shown in Fig. 6, the hysteretic model nicely fits the data of the initial experiment and the optimized experiment. In contrast to this, the simple model either lies below or above the measured data points. Since the residuals of the simple model shown in Fig. 7 follow a distinct pattern, the simple model can be assumed to be inappropriate for the observed measurements. Hence, the preconditions for the lack-of-fit test, which are independent normally distributed residuals with equal variances, are not fulfilled for the simple model. The time series of the simple model as well as the corresponding residual plot clearly show its poor fit. The residuals of the hysteretic model are randomly distributed around zero without showing any noticeable pattern. Also note that the residuals for the substrate curve of the simple model vary in a range of 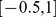, which is by far larger than the corresponding residuals of the hysteretic model. The F-values of the lack-of-fit test for the hysteretic model are 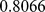 for the substrate curve and 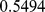 for the product curve. The critical value is given by 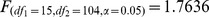 for five independent measurements, each of which consists of 26 measurement points. As the calculated values clearly lie below the critical F-value, the model cannot be rejected. Of course, the values of the lack-of-fit test heavily depend on the measurement data that are being used. So the values presented here should just be considered as exemplary result values of such a test. Multiple repetitions with different randomly disturbed data sets failed to reject the hypothesis, though. Considering the described goodness of fit methods, the hysteretic model is appropriate for the given experimental data.

**Table 5:** The re-estimated parameters for the simple model.

Parameter:	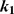	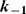	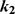
Value:	6.5194	0.0	2.8908

The re-estimated parameters for the simple model on the initial measurement data and measurement data for the identified optimal design. For correct hypotheses it should be possible to determine a parameter set that fits to all available measurements.

**Table 6:** The re-estimated parameters for the hysteretic model.

Parameter:	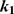	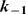	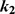	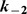	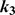	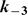	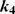	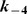	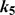	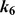
Value:	0.0266	0.0	0.04484	0.0	0.0	5.1121	0.0247	0.0	0.0	4.2822

The re-estimated parameters for the hysteretic model on the initial measurement data and measurement data for the identified optimal design. For correct hypotheses it should be possible to determine a parameter set that fits to all available measurements.

**Figure 8:**
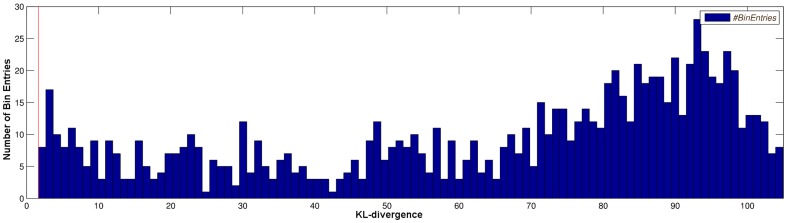
Histogram plot the KL-divergence distribution for 1000 random parameters of the simple model. Only values larger than the red bar are observed. This indicates that no lower distances than the proposed minimal distance of the robust optimal design have to be expected.

**Figure 9:**
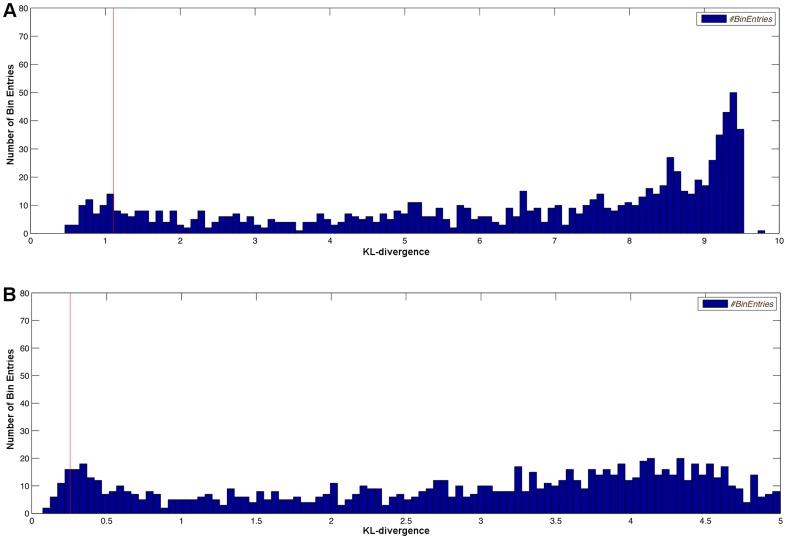
Histogram plot of the KL-divergence distribution of model distances for 1000 random parameters of the simple model. The experimental designs are optimized but lack of robustification of any parameters (A) and additionally lack of perturbations (B). KL-divergence values below the value of the red bar indicate that there are parameter combinations for which the discrimination is worse than the one of the proposed optimal design and decisions might become ambiguous.

To verify that the KL-divergence of 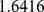 found for the optimal design is the smallest distance that can be observed, a histogram plot is used. 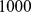 random initializations of parameters of the simple model are performed with respect to the box constraints mentioned above and the KL-divergence values are summarized into 100 equally spaced histogram bins. Some outliers that occurred rarely in the KL-divergence range of 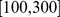 are not shown in Fig. 8. All measured distances are greater or equal to the red bar which indicates the KL-divergence for the worst case parameters. Therefore, the calculated design is able to distinguish between the models at least by this given worst case KL-divergence. Similarly, Fig. 9 shows a histogram plot of the KL-divergences for two experiments that have been optimized without robustification of parameters. Both histograms contain bins with KL-divergences smaller than the proposed KL-divergence of the optimal design indicated by the red bar. Therefore, no conclusions about the actual performance of the calculated experiments can be drawn. This shows the benefit and necessity of the robustification approach, as for robust optimal designs, no parameters can cause a lower discrimination power between the two models than the one observed for the proposed design.

### Benchmark for model discrimination

In this section we apply our tool on a benchmark for model discrimination as presented in [6]. For convenience, the model is briefly summarized. The example models a biochemical reaction network of a fictitious organism with several participating metabolites. The experiment takes place in a tank reactor which is characterized by the inflow 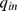 and the outflow 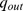 of a substrate with concentration 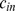.

This reaction network is defined by the following ODEs:
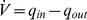
(21)

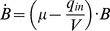
(22)

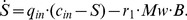
(23)In Eq. (21), 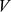 is the volume of the tank reactor. As the flow rates 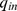 and 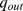 are assumed to be equal throughout the experiment, the volume change of the reactor 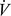 is left out in the following. In Eq. (22) and (23), 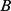 represents the biomass concentration and 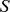 models the substrate concentration, respectively.
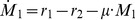
(24)

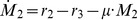
(25)

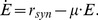
(26)


Directly after the uptake of substrate into the organism, 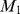 is synthesized and the enzyme 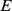 catalyzes the irreversible conversion of 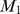 to 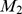. The corresponding ODEs are given in Eq. (24)–(26). The reaction rates 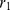, 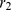 and 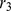 involve Michaelis-Menten kinetics with the parameters given in Tab. 7. For reaction rates 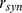 and 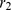 a formal kinetic law is used to model the inhibition of the enzyme activity by the concentration of 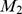. The parameters for these terms are listed in Tab. 7. The different involved kinetic laws suggest that the concentration of 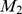 influences both the synthesis and the activity of the enzyme. Eq. (31) models the partial substrate uptake by the organism, which is converted into biomass [6].
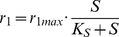
(27)

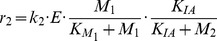
(28)

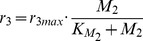
(29)

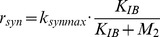
(30)

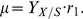
(31)The model described above is the *correct* model, and is used for the generation of *in silico* data as described in the next section. The two different hypothetical models are constructed by differently changing some reaction rate equations. For model A the velocity of the enzyme synthesis is altered to be constant, i.e. 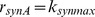. In model B the last fraction of 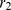 is omitted, which leads to 
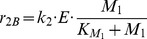
. The conversion of metabolite 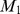 to 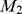 in model A is therefore only regulated by a non-competitive inhibition of the enzyme by 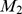. For model B the enzyme synthesis is controlled depending on the concentration of 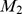. The non-competitive inhibition of the enzyme activity by 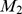 does not play a role for model B anymore, as the corresponding term is left out [6]. Note that in this case both of the considered models are somewhat different from the model used for data generation. Accordingly, the task of the discrimination approach is to figure out which of these models is best suited to approximate the *correct* model. In the following discrimination example, the initial and intermediate values of the flow rates 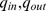 and the substrate concentration 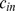, as well as the measurement time points are subject to design. The calculated design is robustified against the parameter 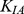 of model A.

**Table 7:** The parameters of the correct model.

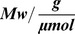	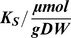	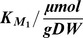	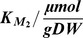	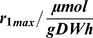	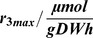
342.3e-6	0.4437	12.2	10.0	2.4e4	3.0e6

The parameters of the correct model as given in [6]. Note that the values of 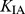 and 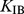 mentioned as correct parameters in [6] are erroneously interchanged.

#### Data generation

As described by Kremling et al., measurements are generated using the correct model with an additional random noise applied to each simulated concentration value using a relative error model [6]. An independent normally distributed random variable 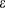 with standard deviation 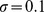 and 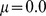 is applied multiplicatively, i.e. each value 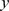 predicted by the correct model is altered to 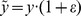. The presented examples use the symmetrized version of Eq. (1) as the objective function, i.e.
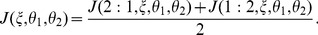
(32)The parameters of the correct model can be found in Tab. 7. In order to keep the presented results comparable to the results of Kremling et al. the model parameters as well as the initial species concentrations are taken from the appendix of [6]. Note that the values of 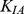 and 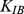 mentioned as correct parameters in [6] are erroneously interchanged. Throughout this section, the values for 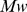, 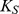, 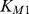, 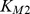, 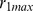 and 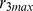 are used for both considered models and are assumed to be known. The remaining parameters 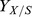, 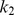, 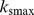, 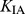 and 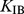 are estimated according to the simulated measuremental data using the method described in [6]. The value of the parameter 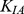 given in Tab. 7 is used as an initial guess for the parameter robustification performed in the inner-loop of the model discrimination algorithm. The final worst-case value for 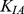 is given separately for each of the presented experimental designs. In Tab. 8 the parameters estimated on the initial experiment are summarized [6].

**Table 8:** Re-estimated parameters for both models on the initial benchmark experiment.

	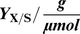	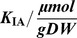	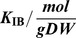	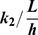	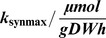
Model A	6.968e-5	0.104	-	5.988e6	7.2e-3
Model B	7.031e-5	-	0.166	5.559e6	8.2e-3

Re-estimated parameters for both models on the initial experiment.

**Figure 10:**
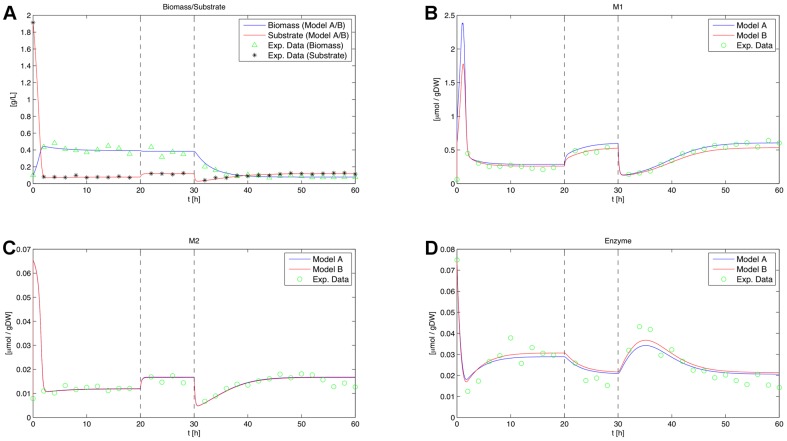
Initial time courses for biomass, substrate, 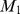
 **,** 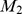 **and the enzyme.** The initial time series for biomass and substrate concentration (A), species 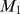 (B), species 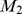 (C) and the enzyme (D) are shown. In image (A) both models lead to the same response. The remaining time courses show only small differences. None of the models clearly fits the data worse. The vertical bars indicate perturbation time points.

For the simulated experiment by Kremling et al. the initial substrate concentration is set to 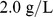 and the initial flow rates are set to 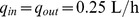. Measurements are taken every two hours which results in thirty measurement points for the experimental duration of 60 hours. After 20 hours both flow rates are raised to 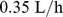 and after 30 hours the substrate concentration is reduced to 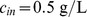. Fig. 10 shows the initial time courses of the two models and illustrates that the models react sensitively on such inflow changes [6]. Additionally, Fig. 10 clearly indicates that both models fit the data equally well. By only taking this experiment into account, a reasonable distinction between the two models would not be possible. Hence, a different experimental design, which is able to directly discard one of the hypothetical models, is desirable. The next sections show the resulting model responses achieved by calculating different input and perturbation profiles for the experiments. In the presented results, usually only the plots for species 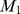 and the enzyme are shown, because all curves of the other species are largely the same for the performed experiments. The design variables are the tank reactor flow rates 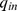 and 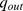, the substrate concentration 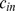, the measurement time points and two perturbations.

As described in [6], an F-test is used to statistically verify the rejection of inappropriate hypotheses. Therefore, the standard deviation of the residuals is calculated for both investigated models. The ratio of the larger value divided by the smaller value follows an F-distribution and can be compared to the critical value of an F-distribution with corresponding degrees of freedom. Here, the degrees of freedom are the amount of measurements or rather the amount of residuals. Considering for example the standard deviations of the absolute values of the enzyme residuals for the initial experiment 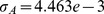 and 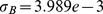. The critical value for e.g. thirty measurements and a significance level of 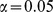 is given by:
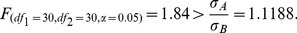
(33)In this case the test fails to reject the null hypothesis that the two standard deviations are not significantly different and none of the models can be discarded for the initial experiment. If this ratio would be larger than the critical value the null hypothesis is rejected and the residual standard deviation of model B would be significantly smaller. Note that in this case the residuals are also calculated at perturbation positions, which is the reason they are also plotted in the presented figures.

#### Results and discussion

The calculation of a robust optimal design presented in this section differs from the approaches carried out by Kremling et al. by additionally allowing an optimization of the measurement and perturbation time points. The substrate concentration 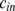 is set to 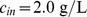 and the initial flow rate is set to 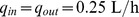. At the 10th time point the flow rates are increased to 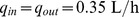 and at the 15th time point the substrate concentration is decreased to 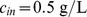. As the measurements are initially performed every two hours, this initial design equals the one described by Kremling et al. in Tab. 1 of [6]. The initial flow rates are restricted to 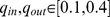 and the initial substrate concentration is restricted to 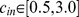. The intermediate flow rate changes are restricted to 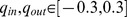 and the intermediate substrate concentration change is restricted to 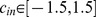. The parameter 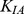 is subject to robustification and constrained to the interval 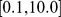. The parameters for the step function for prevention of multiple measurements are set to a width of 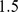 with the center located at 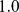. For the second step function the parameters are set to a width of 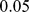 with the center at 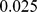. The integration tolerances and sensitivities are set to 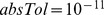 and 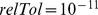.

**Figure 11:**
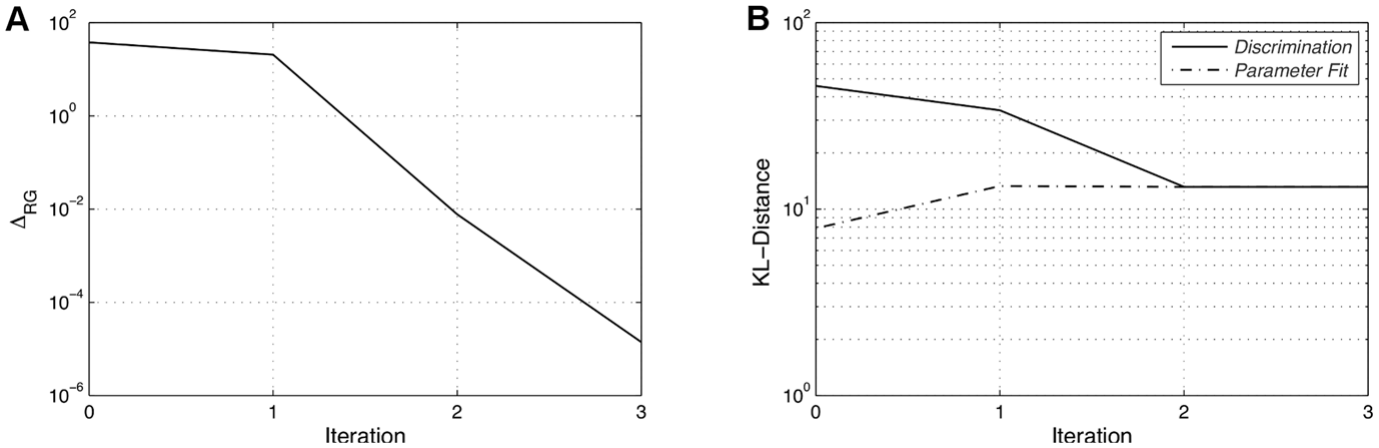
Robustification gaps and objective values for the second experiment. The robustification gap 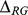 is displayed in (A), whereas (B) shows the corresponding objective values of the respective new designs (solid line) and the objective values of this design with re-estimated worst case parameters (dashed line). All values are plotted versus the corresponding iteration number.

As shown in Fig. 11, the optimal design is found after three iterations of the discrimination algorithm. The homotopy [10] is started after the robustification gap drops below 0.1 in the second iteration and it is performed once until the stopping criterion of 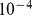 is reached. The final KL-divergence value is 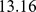 for the calculated design with 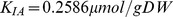. Calculating the robust optimal design including five parameter fits after each discrimination run took approximately 7.5 hours on one core of an Intel Xeon X5460 CPU with 3.16 GHz.

As in the previous example, the initially estimated parameters do not fit multiple measurements of different experimental designs. Therefore, the parameters are fitted simultaneously to measurements of the initial design and the new design. In Tab. 9 the re-estimated parameters for both models are shown. For model A the parameters are re-estimated 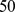 times with random initializations to avoid locally optimal parameters. The best parameter set is listed in the table. The necessity for parameter re-estimation was also mentioned by Kremling et al. where new model parameters had to be determined after each calculation of new designs. Obviously, if a model is correct, it should be possible to find a parameter set that is suited to fit several distinct measurements produced by the underlying biochemical process. If no parameters can be found to simultaneously fit the observed data, the model might be inappropriate.

**Table 9:** Re-estimated parameters for both models on the benchmark experiment.

	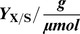	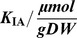	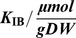	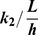	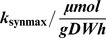
Model A	7.3093e-05	0.01965	-	9.4370e6	0.006924
Model B	7.3094e-5	-	0.0081	6.7968e6	0.0184

Re-estimated parameters for both models on the benchmark experiment.

**Figure 12:**
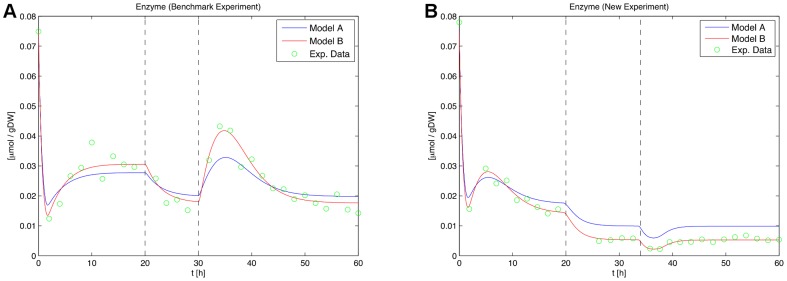
Time courses of the enzyme for the initial and the new design for the second experiment. Trajectories of the enzyme for the benchmark experiment (left) and the new experiment (right) with re-estimated parameters are shown. Model A clearly fits the data worse in both cases. The vertical bars indicate perturbation time points.

**Figure 13:**
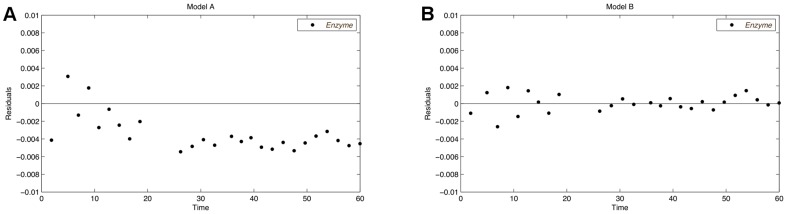
Residual plots after discrimination with re-estimated parameters for the second experiment. The results for model A (A) and for model B (B) are shown. The residuals of model A show a clear trend, which indicates a bad fit of the model.

Fig. 12 shows the time courses of both models for the enzyme with the re-estimated parameters. In both cases, model A fits the measurement data worse. The initial flow rate of the new design is raised to 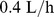 and the initial substrate concentration is lowered to 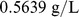. The flow rate change remains at 20 hours and it is raised from 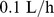 to 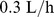. The substrate concentration change is raised from 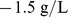 to 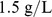 and applied after 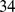 hours. Two measurement points are removed and the measurements are moved into regions where the largest model distances are observed. The stimulation time points of the new experiment are quite similar to the initial time points. This indicates that the initial perturbation points are already a good choice. Consequently, the optimization of the stimulation time points could not be exploited for an enlarged model distances in this case. Instead, the discrimination power is raised by rearranging measurement points and increasing the stimulations. The residual plots of the enzyme time courses for measurements of the new experiment are shown in Fig. 13 and clearly support the inappropriateness of model A.

**Figure 14:**
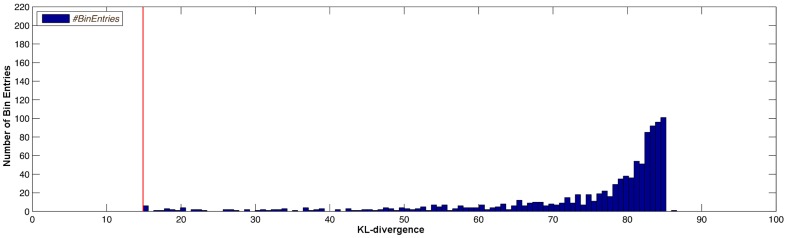
Histogram plot of the KL-divergence distribution for the second experiment. Distribution of the KL-divergence for different values of 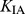, randomly distributed in the interval [0.005; 10.0]. The red line represents the KL-divergence achieved by the worst-case parameter for this design.

**Figure 15:**
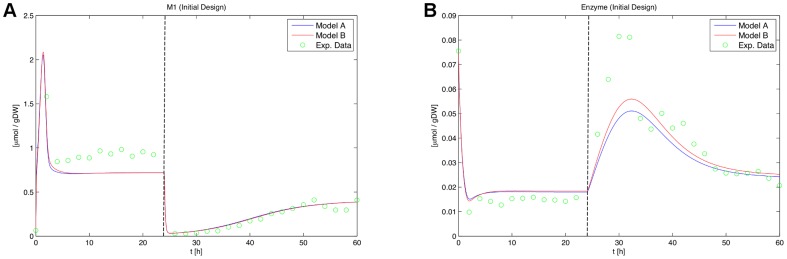
Time courses of the optimal design proposed in *Large Steps on the Inputs* by Kremling et al. after re-estimation of parameter 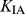
 **.** The 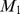 (A) and enzyme concentration (B) time courses for the optimal design proposed by Kremling et al. are shown. Upon re-estimation of the parameter 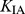, the model responses become quite similar, which shows the necessity of the robustification approach. The vertical bars indicate perturbation time points.

The standard deviation of the enzyme residuals for measurements of the new experiment are 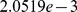 and 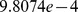 for model A and model B, respectively. As two measurement time points are omitted in the new design and two are used for perturbations, only 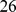 degrees of freedom are used for the following F-distribution:
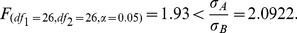
(34)The null hypothesis is rejected, which indicates that the residuals of model B have a significantly smaller standard deviation compared to the residuals of model A. Similar to the example presented before, the histogram plot shown in Fig. 14 is based on 1000 random values of the parameter 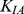 and confirms the robustness of the design with respect to this parameter set. Some rarely occurring outliers above 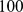 are removed to improve the depiction. The red bar indicates the lowest observable KL-distance and the actually observed values might be significantly larger in real experiments for the correct parameter values. To exemplarily show the lack of robustification presented in section *Large steps on the inputs* by Kremling et al., the parameter 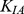 is re-estimated on simulated measurement data of the proposed optimal design. Fig. 15 shows the result of this re-estimation. It is obviously impossible to draw a reasonable conclusion which model fits the data worse in this case.

An issue that frequently occurs during the calculation of robust optimal designs is related to the constraints of the flow rates. Kremling et al. used 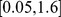 as constraints for the flow rates, which does not work well in the presented discrimination attempt. It is also doubtful if such large flow rates are practically meaningful. In our example, the flow rates are constrained to significantly smaller ranges. Using the graphical user interface, the model behavior upon such value changes can easily be investigated. For example raising the flow rate from 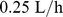 to a value of 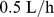 results in an increased 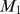 concentration by factor 300 (data not shown). Of course, the corresponding derivatives get even larger and their calculation may cause numerical problems. In trials with larger flow rate intervals, usually the parameter re-estimation still works fine, but during the calculation of a discriminating design, the objective function grows enormously. Ipopt converges to a point of local infeasibility and is finally unable to sufficiently reduce the dual infeasibility. This results in an interrupted optimization after a failed restoration phase. To partially get rid of these problems it helps to reduce the allowed variation of the flow rates and to add the Ipopt settings listed in Tab. 10. Particularly, if input profiles contain perturbations that are already set to the maximum value of the feasible range, the optimization starting point is close to the bounds. In this case Ipopt relaxes the bounds which can result in an infeasible starting point. The Ipopt settings listed in Tab. 10 avoid such a boundary relaxation and make the initial point stay feasible. Additionally, the Ipopt convergence tolerance *tol* is increased to 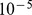 in order to speed up the calculations and avoid the *Solved to Acceptable Level* warning. Of course, a reduction of the integrator tolerances and sensitivities might also be possible, but would in turn raise the computation time.

**Table 10:** Ipopt settings for the benchmark experiment.

Parameter Name	Type	Value
*tol*	double	10^−5^
*bound_push*	double	10^−99^
*bound_frac*	double	10^−99^
*slack_bound_push*	double	10^−99^
*slack_bound_frac*	double	10^−99^
*bound_relax_factor*	double	0.0

Additional Ipopt settings for the benchmark experiment.

## Conclusions

In this contribution, the theoretical and practical concepts that are used for the implementation and application of a convenient software tool for the purpose of robust optimal design of experiments for model discrimination, are presented. It is shown how a modeling approach based on ordinary differential equations can be combined with numerical optimization to calculate new experimental designs that allow to select between several rival models. We demonstrate the relevance of the robustification approach as well as the functionality and usefulness of the ModelDiscriminationToolkitGUI, by applying the software on two artificial biochemical reaction networks. Our approach successfully identifies inappropriate hypotheses with respect to various experimental design constraints. The goodness of fit of the models is evaluated using residual plots and a lack-of-fit test. The robustness of the given designs against parameter changes is verified by use of a KL-divergence histogram of numerous random initialized parameters. The major benefit of the graphical user interface is the ability to interactively investigate the model behavior and optimization strategies. Additionally, the GUI facilitates data handling and keeps the numerous experimental design variables clearly arranged. A general recipe for the calculation of robust optimal experimental designs, however, cannot be given on the basis of the presented examples. It usually makes sense to evaluate different discrimination scenarios, e.g. using the ModelDiscriminationToolkitGUI. The use of reasonable ranges for the involved perturbations, to avoid time courses that are unlikely for an observed biochemical phenomenon, combined with a sophisticated arrangement of the measurement points seems to be a good initial choice, though. The software is licensed under the GNU General Public License and freely available for download at http://sourceforge.net/projects/mdtgui/. In future releases, the ModelDiscriminationToolkitGUI could be extended by a formula parser, e.g. using the well known SMBL format, to further facilitate the investigation of new models and allow to access numerous reviewed biochemical models that are available online [32].
